# Formation and Release Enhancement of a Novel Small-Molecule Hydrogel Containing Sulindac and Meglumine

**DOI:** 10.3390/gels12030198

**Published:** 2026-02-27

**Authors:** Jiaxin Chen, Baimin Niu, Huizhen Sun, Weitao Fang, Mingjun Li, Xinru Lu, Jue Wang, Jiawei Han, Xiaoqian Liu

**Affiliations:** School of Pharmacy & School of Biological and Food Engineering, Changzhou University, Changzhou 213164, China; mschenjx@126.com (J.C.); 18761327921@163.com (B.N.); 18014688935@163.com (H.S.); fangweitao3910@163.com (W.F.); limingjun615@163.com (M.L.); 14752109014@163.com (X.L.); wangjue@cczu.edu.cn (J.W.)

**Keywords:** sulindac, meglumine, hydrogel, solubilization, complexation

## Abstract

Up to now, most hydrogel-related studies have been devoted to the preparation of drug-containing macromolecular gels via the introduction of polymer matrices, together with the clarification of their assembly mechanisms and biomedical applications. In contrast, studies concerning the design of small-molecule gel systems remain relatively limited. As gel research progresses, drug small-molecule hydrogels have attracted growing interest for formulation development. This study investigated whether designing a small-molecule hydrogel could serve as an effective solubilization approach for sulindac (SUL)—a nonsteroidal anti-inflammatory drug clinically restricted by its poor aqueous solubility. Then, a SUL small-molecule hydrogel was prepared by straightforward mixing of SUL with biologically safe meglumine (MEG) in a minimal volume of deionized water, which exhibited a characteristic three-dimensional network structure and favorable viscoelastic properties. The characterization and simulation results indicated that the hydrogel formation was contingent upon the SUL-MEG miscibility, dissolution-aggregation equilibrium and intermolecular self-assembly. Consequently, the resulting SUL-MEG hydrogel exhibited 546 times higher solubility compared to the pure SUL. Meanwhile, the SUL-MEG hydrogel demonstrated superior release kinetics and supersaturation capacity, characterized by rapid attainment of peak concentrations and sustained supersaturated release. These enhanced performances were attributed to the high-energy state of the hydrogel itself and the molecular complexation between SUL and MEG. In conclusion, this study presents a feasible formulation strategy for overcoming the poor water solubility of insoluble drugs through the development of small-molecule hydrogel formulations.

## 1. Introduction

In the development of pharmaceutical formulations, it has been noted that certain small-molecule crystalline drugs (e.g., clarithromycin [1] and lenvatinib mesylate [2]) and amorphous drugs (e.g., amorphous lurasidone hydrochloride [3] and amorphous curcumin [4]) are prone to aggregating into viscous gel-like aggregates. Namely, the organic small-molecule gels were formed during manufacturing or dissolution procedures. For example, upon contact with the dissolution medium, amorphous curcumin rapidly aggregates to form a viscous and solid-like gel, which ultimately led to poorer dissolution performance relative to its crystalline counterpart [4]. With regard to the development of solid dosage forms for lenvatinib mesylate, gelation took place both during wet granulation after mixing with excipients and throughout the dissolution process [2]. These phenomena not only pose considerable challenges to the development of formulation compositions and manufacturing processes, but also lead to a marked retardation of drug release as a result of gel formation, consequently compromising the in vivo drug absorption and clinical therapeutic effectiveness.

Gels constitute a unique state of matter lying between solids and liquids, consisting of cross-linked networks formed by colloidal particles or polymer molecules [5,6,7]. Gelation refers to the process wherein molecules assemble into such networks under specific conditions, with the involved compounds designated as gelators. Based on their molecular composition, gels are categorized into polymer gels and small-molecule gels. To date, polymer gels (e.g., poloxamer, chitosan and carbomer) have found extensive applications in pharmaceuticals, food packaging and industrial chemistry [8,9,10]. In recent years, small-molecule gels have emerged as a prominent research focus [11,12]. Compared with polymeric gels, self-assembled small-molecule gels exhibit theoretical advantages such as enhanced biodegradability and biocompatibility, endowing them with considerable application potential in fields such as tissue engineering and pharmaceutical engineering. However, these anticipated advantages have not yet been realized as initially reported [1,2,3,4]. Given that current studies suggest single-component small-molecule gels are not an optimal solubilization strategy, could binary small-molecule gel systems serve as a feasible alternative for modulating the physicochemical properties of insoluble drugs?

In this study, sulindac (SUL) is an important non-steroidal anti-inflammatory drug classified as a benzothiazine-derived compound [13,14]. As a prodrug, SUL undergoes in vivo metabolism into an active sulfide metabolite for the inhibition of cyclooxygenase following oral administration, thereby decreasing prostaglandin biosynthesis and exerting anti-inflammatory, analgesic and antipyretic activities. Clinically, SUL is formulated as oral tablets and capsules with a relatively broad spectrum of indications. It is frequently used in the management of osteoarthritis, as it effectively alleviates symptoms including joint pain, stiffness and impaired mobility [13,14,15,16]. Nevertheless, owing to its chemical structure containing hydrophobic aromatic and heterocyclic groups, SUL is classified as a biopharmaceutics classification system (BCS) Class II drug with low aqueous solubility, which restricts its release in the gastrointestinal tract and consequently compromises oral bioavailability. In addition to SUL, meglumine (MEG) is a pharmaceutically acceptable solubilizing excipient endorsed by international regulatory guidelines, and it has been extensively incorporated into solid oral dosage forms and parenteral formulations to enhance drug absorption [17,18,19]. Accordingly, this study sought to validate the hypothesis that designing small-molecule hydrogels represents a viable strategy for optimizing the physicochemical properties of SUL. Through the incorporation of biocompatible MEG, a binary SUL small-molecule hydrogel (i.e., SUL-MEG hydrogel) was designed to investigate the gelation mechanism, improve the solubility of SUL and its release profile, and ultimately optimize its drug performance.

## 2. Results and Discussion

### 2.1. Theoretical Miscibility of SUL and MEG

The Hansen solubility parameter (δ), a robust analytical tool for assessing component compatibility in multi-component systems, has been extensively adopted for component screening and interaction prediction throughout pharmaceutical formulation development [20,21]. According to the miscibility criterion, when the solubility parameter discrepancy (Δδ) between components is less than 7 MPa^1/2^, the thermodynamic driving force generated by intermolecular interactions within the system is adequate to overcome the energy barrier related to mixing, thereby facilitating the formation of a homogeneous component system. In contrast, a Δδ value beyond this critical cutoff indicates an intrinsic risk of phase separation in the system. As presented in Table S1, the δ values of SUL and MEG were measured to be 23.74 MPa^1/2^ and 25.89 MPa^1/2^, respectively. The calculated Δδ between SUL and MEG was 2.15 MPa^1/2^, which was well below the 7.0 MPa^1/2^ threshold, suggesting a high likelihood of favorable miscibility between these two substances.

### 2.2. Formation of SUL-MEG Hydrogel

**Figure 1:**
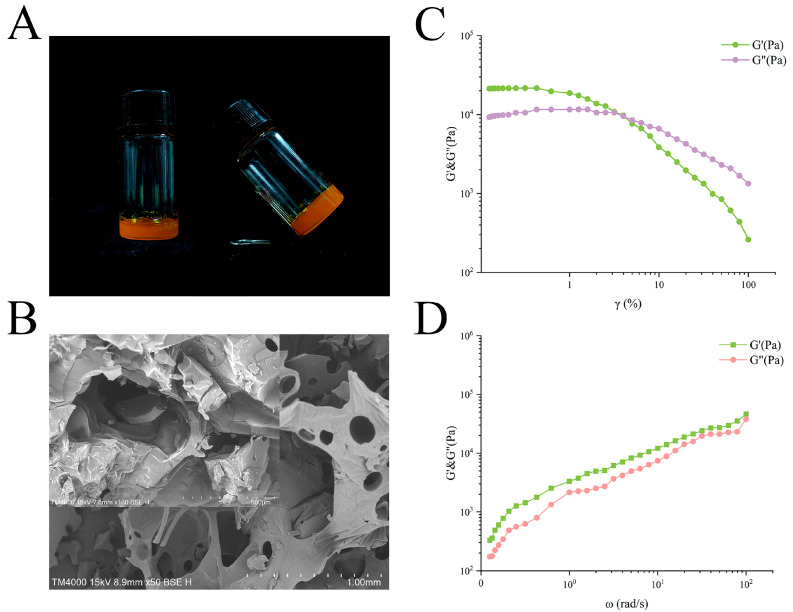
(**A**) Photograph and (**B**) SEM image of SUL-MEG hydrogel. (**C**) Strain sweep and (**D**) frequency sweep of SUL-MEG hydrogel.

When SUL and MEG were individually blended in deionized water, the resulting product showed an orange color along with viscoelastic properties (Figure 1A). No noticeable fluidity was observed in the samples when the glass container was tilted. Scanning electron microscopy (SEM) analysis revealed that the SUL-MEG hydrogel featured a porous three-dimensional network structure, which was composed of stacked irregularly folded lamellae (Figure 1B). The irregular block was likely attributable to the gold plating and heating treatments during the SEM pretreatment process. In addition, the microscopic morphology of the SUL-MEG hydrogel was further examined using high-resolution microscopy under cryogenic conditions, which also demonstrated its well-defined three-dimensional network architecture (Figure S1).

The intrinsic formation mechanism of this unique morphology was presumably due to the self-assembly and crosslinking of gelators, triggered by intermolecular interactions between SUL and MEG. Notably, the three-dimensional network of the hydrogel contained densely distributed pores that could efficiently trap water molecules via surface tension and capillary action, thereby facilitating the formation of a stable hydrogel system. Additionally, the SUL-MEG hydrogel retained its viscosity after 30 days of storage at 25 °C, indicating excellent long-term stability of its three-dimensional structure (Table S2). In essence, a basic prerequisite for gel formation is the establishment of dynamic equilibrium between the dissolved and aggregated states of gelators in the solvent [2,4]. Experimental results suggested that the presence of MEG likely improved the aqueous solubility of SUL, thus effectively regulating the establishment of the molecular dissolution-aggregation equilibrium.

### 2.3. Rheological Analysis of SUL-MEG Hydrogel

Rheological analysis offers a crucial understanding of gel structures by assessing essential characteristics such as flow behavior and rigidity. The elastic modulus (G′), also known as the storage modulus, measures a material’s capacity for elastic recovery, while the viscosity modulus (G″) refers to the loss modulus and quantifies its viscous energy dissipation. Together, G′ and G″ act as core indicators for evaluating the dynamic mechanical behavior of hydrogels subjected to oscillatory shear. A gel state is defined by G′ surpassing G″, whereas the solution state is characterized by G″ predominating over G′ [22].

The dynamic oscillatory rheological test was performed to characterize the viscoelastic property of SUL-MEG hydrogel. Both G′ and G″ maintained stability at strain amplitudes below 3%, indicating that the network structure resided within the linear viscoelastic region (Figure 1C) [23,24]. Beyond this 3% strain threshold, a significant decrease in modulus values was observed, accompanied by a reversal of viscoelastic behavior (G″ > G′), acting as a phenomenon indicative of three-dimensional network disruption. When the critical strain value was exceeded, it led to modulus inversion with the system displaying fluid-like characteristics. Such viscoelastic behaviors are typical of materials with prominent viscoelasticity. The shear-induced transition in viscoelastic response was presumably associated with the dissociation of non-covalent interactions (e.g., hydrogen bonds, van der Waals forces) within the hydrogel, which ultimately resulted in the breakdown of its three-dimensional network structure [25].

To further investigate the viscoelastic response of the SUL-MEG hydrogel, a frequency sweep test was conducted under conditions of 0.1~100 rad/s angular frequency and 3% strain (within the linear viscoelastic region). As illustrated in Figure 1D, both G′ and G″ of the SUL-MEG hydrogel increased concurrently with rising angular frequency, which might be attributed to synergistic coupling effects between SUL-MEG molecular chains. Consequently, the SUL-MEG hydrogel exhibited elastic-dominant behavior (G′ > G″), and its favorable rheological property laid a theoretical foundation for potential applications in drug delivery systems.

### 2.4. Characterization of SUL-MEG Xerogel

**Figure 2:**
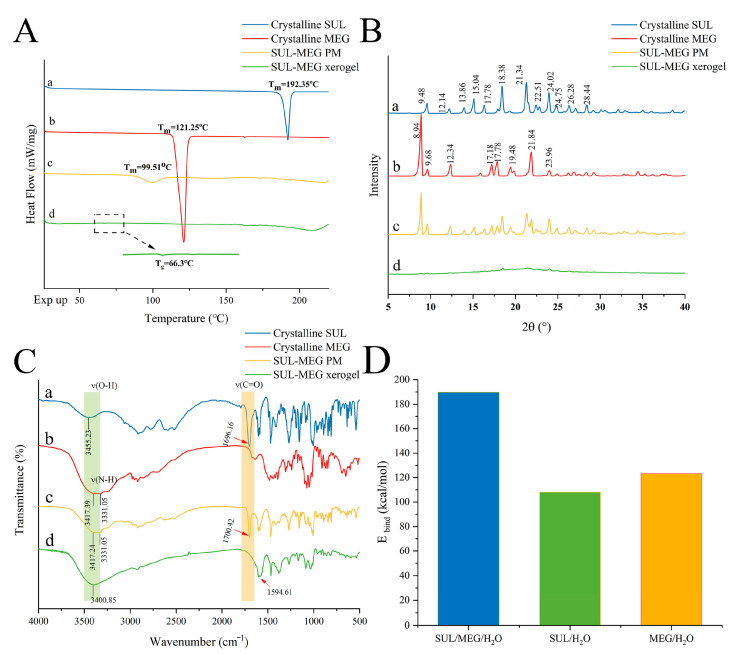
(**A**) DSC thermograms, (**B**) PXRD patterns and (**C**) FTIR spectra of (a) crystalline SUL, (b) crystalline MEG, (c) SUL-MEG PM and (d) SUL-MEG xerogel. (**D**) E_bind_s between components in the SUL-MEG-H_2_O system.

Firstly, differential scanning calorimetry (DSC) was utilized to examine the thermal characteristics of the SUL-MEG xerogel (Figure 2A). As indicated by the DSC data, crystalline SUL and MEG displayed typical endothermic melting temperatures (T_m_) at 192.35 °C and 121.25 °C, respectively (Figure 2A(a,b)). Owing to the eutectic effect occurring during heating, the endothermic melting peak of the SUL-MEG physical mixture (i.e., SUL-MEG PM) underwent a reduction and shift to 99.51 °C (Figure 2A(c)). In contrast, the SUL-MEG xerogel lacked the endothermic peaks corresponding to the two pristine components and presented a single glass transition temperature (T_g_) at 66.30 °C (Figure 2A(d)), suggesting the formation of a single-phase amorphous structure with random molecular arrangement in the xerogel during gelation [26].

Additionally, powder X-ray diffractometry (PXRD) was used to assess crystallinity changes in the SUL-MEG xerogel (Figure 2B). The lack of crystallinity is identifiable via the appearance of an amorphous halo in PXRD patterns. Experimental findings demonstrated that crystalline SUL displayed characteristic diffraction peaks at 9.48°, 12.14°, 13.86°, 15.04°, 17.78°, 18.38°, 21.34°, 22.51°, 24.02°, 24.75°, 26.28°, and 28.44° (Figure 2B(a)). Crystalline MEG exhibited numerous characteristic peaks between 8° and 30° (Figure 2B(b)). The PXRD pattern of the SUL-MEG PM showed a superposition of diffraction peaks from the respective crystalline constituents (Figure 2B(c)). Conversely, the PXRD pattern of the SUL-MEG xerogel displayed a marked reduction in crystalline diffraction peaks (Figure 2B(d)), further verifying its nearly amorphous nature.

Moreover, Fourier transform infrared spectroscopy (FTIR) was employed to explore intermolecular interactions in the SUL-MEG xerogel (Figure 2C). Crystalline SUL displayed a typical vibrational peak for the hydroxyl group (-OH) at 3455.23 cm^−1^, whereas the carbonyl (C=O) vibrational peak of its carboxylic acid group (-COOH) was observed at 1696.16 cm^−1^ (Figure 2C(a)). For crystalline MEG, vibrational peaks associated with the hydroxyl (-OH) and amino (-NH) groups were detected at 3417.39 cm^−1^ and 3331.05 cm^−1^, respectively (Figure 2C(b)). The FTIR spectrum of SUL-MEG PM exhibited a straightforward superposition of characteristic peaks from both components (Figure 2C(c)). Following the formation of the SUL-MEG xerogel, the free -OH and C=O peaks of SUL disappeared, and an asymmetric stretching peak for -COO^−^ emerged at approximately 1594.61 cm^−1^. Simultaneously, the original -OH and -NH peaks of MEG showed a shift and broadening centered at ~3400.85 cm^−1^ (Figure 2C(d)), which might be due to proton transfer (between the -COO^−^ group of SUL and the -NH_2_^+^ moieties of MEG) and/or hydrogen bond interactions (involving the C=O group of SUL and -OH group of MEG).

### 2.5. Formation Mechanism of SUL-MEG Hydrogel by E_bind_ Calculation

Based on the aforementioned experimental findings, a critical question emerged: why could individual SUL or MEG components not spontaneously form hydrogels in water, while their combination successfully yielded a stable hydrogel? To resolve this conundrum, this study employed molecular dynamics (MD) simulations to elucidate the formation mechanism of the SUL-MEG hydrogel at the molecular interaction level. Specifically, MD simulations revealed an inherent correlation between binding energy (E_bind_) and gelation. As shown in Figure 2D and Table S3, the E_bind_ of the SUL-MEG-H_2_O ternary system was 189.518 kcal/mol. A comparison of E_bind_ values between the ternary system (SUL-MEG-H_2_O) and binary systems (SUL-H_2_O, MEG-H_2_O) demonstrated that the hydrogel system exhibited a significantly higher E_bind_ than the individual components interacting with water. Generally, higher E_bind_ indicates stronger intermolecular forces, which facilitate the formation of stable homogeneous systems [20,21,26,27]. The E_bind_ calculation suggested that the synergistic interactions among the three components in the hydrogel exceeded the forces between each component and water. Therefore, it could be inferred that the formation of the SUL-MEG hydrogel stemmed not only from the favorable miscibility of SUL and MEG, but more importantly, from their synergistic intermolecular forces that drove the crosslinking and self-assembly of three-dimensional network structures. Moreover, the high E_bind_ characteristic of the SUL-MEG hydrogel system not only explained the thermodynamic driving force for gel formation in this study but also predicted its excellent physical stability.

**Figure 3:**
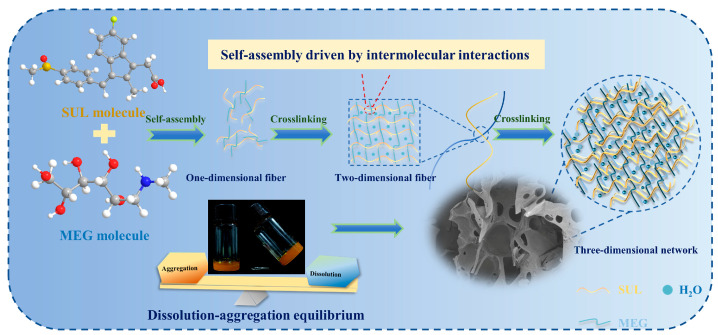
Schematic illustration depicting the formation mechanism of SUL-MEG hydrogel.

Integrating the analytical results of SEM, FTIR and E_bind_, the gelation mechanism of the SUL-MEG hydrogel was proposed with a visual illustration presented in Figure 3. Theoretically, hydrogel formation is governed by a balance between molecular dissolution and aggregation [28,29], requiring both high molecular hydration and effective assembly. Initially, SUL and MEG molecules dissolved upon hydration. As the concentration increased, the dissolved molecules self-assembled into one-dimensional gel fibers via intermolecular hydrogen bonding and/or ionic interactions. These fibers are then further assembled into two-dimensional structures, trapping water molecules through capillary action and surface tension. Ultimately, the two-dimensional structures entangle to form the three-dimensional gel network.

### 2.6. Apparent Solubility of SUL-MEG Hydrogel

**Figure 4:**
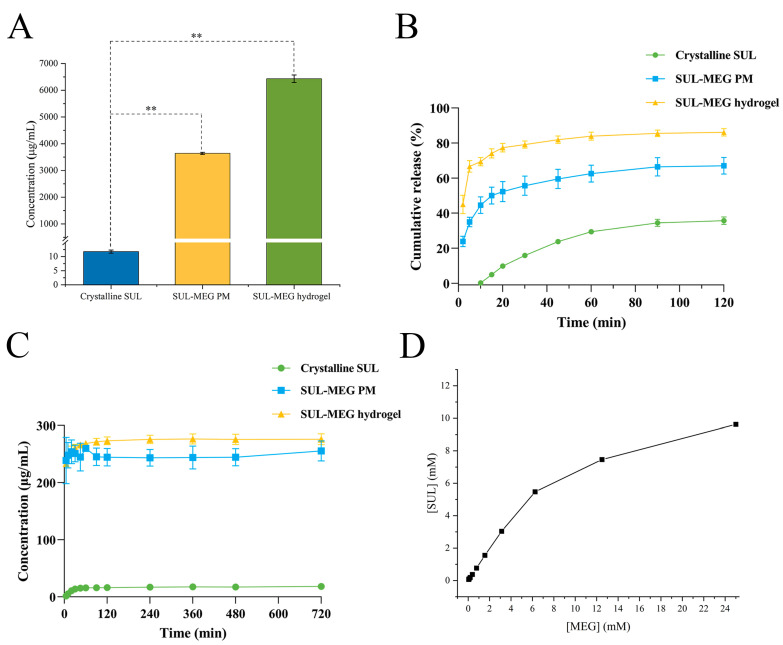
(**A**) Apparent solubility, (**B**) cumulative release profiles and (**C**) supersaturated release profiles of crystalline SUL, SUL-MEG PM and SUL-MEG hydrogel. (**D**) Phase-solubility diagrams of the SUL-MEG system in water. Data were expressed as mean ± SD, *n* = 3. ** *p* < 0.01, compared with crystalline SUL.

The poor aqueous solubility of the drug severely impairs its absorption. Subsequently, the effect of the novel SUL-MEG hydrogel on the aqueous solubility of SUL was assessed via the determination of its apparent solubility (Figure 4A). Experimental results indicated that the equilibrium solubility of crystalline SUL in water at 37 °C was only 11.77 μg/mL. By comparison, the solubility of SUL-MEG PM rose to 3640.24 μg/mL, representing a 309.28-fold enhancement (*p* < 0.01). This solubility improvement was presumably linked to enhanced intermolecular interactions within the solution. Additional studies showed that the solubility of the SUL-MEG hydrogel (6432.13 μg/mL) was remarkably elevated by 546.48-fold relative to crystalline SUL (*p* < 0.01). These findings suggested that the significant enhancement of SUL solubility in the SUL-MEG hydrogel originated not only from the interactions between SUL and MEG in the medium but also was closely associated with the amorphous state generated during the gelation process. The disordered molecular arrangement of the amorphous state effectively reduced the lattice energy barrier through its high-entropy, high-enthalpy, and high-Gibbs free energy properties, thereby promoting drug dissolution [30,31].

### 2.7. Release Performance of SUL-MEG Hydrogel

The cumulative drug release behavior of SUL-MEG hydrogel was assessed under the sink condition (Figure 4B). Release data showed that crystalline SUL only released 9.82% of the drug in the first 20 min, while the cumulative release percentages of SUL-MEG PM and SUL-MEG hydrogel attained 52.31% and 77.41%, respectively. During the entire dissolution process, the cumulative release of SUL-MEG hydrogel was consistently significantly higher than that of SUL-MEG PM and crystalline SUL. At 120 min, the cumulative release of SUL-MEG hydrogel reached 86.18%, which was substantially higher than the 67.05% of SUL-MEG PM and 35.75% of crystalline SUL. Thus, the cumulative release assay demonstrated that the formed SUL-MEG hydrogel system remarkably promoted SUL release under the sink condition. Meanwhile, the improved dissolution performance of the physical mixture could be ascribed to strengthened intermolecular interactions in the solution. In addition to such interactions, the superior release efficiency of SUL-MEG hydrogel was probably related to the high-energy nature of its amorphous form, which lowered the energy barrier for drug release from the matrix.

Furthermore, the supersaturated release capacity and maintenance duration of SUL-MEG hydrogel were examined through non-sink dissolution testing (Figure 4C). For crystalline SUL, the drug concentration released at 120 min was merely 16.30 μg/mL, while those of SUL-MEG PM and SUL-MEG hydrogel rose to 244.60 μg/mL and 273.34 μg/mL, respectively. With the elapse of time, all samples gradually approached their saturation concentrations. At 720 min, the concentration of SUL-MEG hydrogel reached 276.17 μg/mL, representing 15.11-fold and 1.08-fold increases compared with crystalline SUL (18.28 μg/mL) and SUL-MEG PM (255.57 μg/mL), respectively. In addition, the other release test was also conducted for crystalline SUL and SUL-MEG hydrogel in a pH 1.2 HCl buffer to simulate the dissolution behavior of artificial gastric juice. Similarly, SUL-MEG hydrogel performed superior release behavior with high and stable supersaturated concentration. In 720 min, the SUL concentration of SUL-MEG hydrogel reached 46.02 μg/mL and showed a 22.67-fold improvement in crystalline SUL, which could facilitate the absorption of SUL in the gastrointestinal tract. The finding demonstrated that the release profile of the SUL-MEG hydrogel system complied with the “spring-plateau effect” characteristic [21], which inhibited the recrystallization of dissolved SUL in water, significantly extended the duration of supersaturated drug release, and enhanced the sustained supersaturation level.

### 2.8. Solubilization Mechanism of SUL-MEG Hydrogel

Based on the findings of solubility and in vitro release studies, in addition to the SUL-MEG hydrogel, SUL-MEG PM also markedly improved the release concentration of SUL under the supersaturation condition. Accordingly, a phase solubility experiment was performed to explore the underlying solubilization mechanism [32,33,34]. When evaluating the effect of MEG concentration on SUL solubility, a non-linear increase in the aqueous solubility of SUL was noted as MEG concentration increased (Figure 4D). This phenomenon was consistent with the 1:1 A_N_-type complexation mechanism, with a negative solubility deviation observed in the high MEG concentration range [35,36]. The 1:1 A_N_-type complexation refers to the reversible, non-covalent association of one electron acceptor and one electron donor in a strict 1:1 molar ratio, driven by charge-transfer interactions, hydrogen bonding, π-π stacking, or electrostatic forces, to form a discrete binary supramolecular complex. For the 1:1 binary system consisting of SUL and MEG, the equilibrium process could be described by Equations (1)–(5). In the 1:1 A_N_-type complexation system, the equilibrium reaction between SUL and MEG was described as follows [37]:

(1) $${SUL-MEG}_{hydrogel}⇄{SUL}_{solution}+{MEG}_{solution}$$

(2) $${SUL}_{solution}+{MEG}_{solution}\overset{K_{1:1}}{⇄}{SUL-MEG}_{solution}$$

The complexation constant (K_1:1_) for the 1:1 complex formed in solution was

(3) $$K_{1:1}=\frac{\left[SUL-MEG\right]}{\left[SUL\right][MEG]}$$

For the mass balances for SUL and MEG in solution, the concentration can be expressed in terms of known quantities.

(4) $${\left[SUL\right]}_{T}=\left[SUL\right]+[SUL-MEG]$$

(5) $${\left[MEG\right]}_{T}=\left[MEG\right]+[SUL-MEG]$$

where [SUL]_T_ and [MEG]_T_ were the total concentrations of SUL and MEG in solution. Based on Equations (3)–(5), the solubility of the single component SUL was given by

(6) $$[{SUL]}_{T}={\left[SUL\right]}_{o}\frac{K_{1:1}{\left[SUL\right]}_{o}{\left[MEG\right]}_{T}}{{1+K}_{1:1}{\left[SUL\right]}_{o}}$$

Herein, [SUL]_o_ denotes the intrinsic solubility of crystalline SUL in MEG-free solutions, and [MEG]_o_ is the intrinsic solubility of MEG in the absence of SUL. In accordance with the stoichiometric correlations illustrated by the aforementioned equations, the solubility of SUL increased linearly with rising MEG concentration within the range of 0.048 mM to 6.25 mM, a trend that was in good agreement with the experimental phase solubility results (Figure 4D). However, a typical A_N_-type negative deviation appeared in the solubility curve once the MEG concentration surpassed 6.25 mM. This phenomenon was postulated to be linked to the aggregation of free MEG molecules under the high-concentration condition. Such aggregation lowered the effective concentration of free MEG in the solution and consequently weakened the complexation interaction between SUL and MEG [38,39,40].

In order to further explore the essence of the complexation effect, the dissociation possibilities of SUL and MEG were analyzed in detail (Table S4). Firstly, the pH values of deionized water were measured before and after the supersaturated release of crystalline SUL and SUL-MEG hydrogel (Table S5). The initial pH value of deionized water was 7.08 ± 0.01. After the release of crystalline SUL, the pH value of the medium changed to 6.56 ± 0.21. Meanwhile, the pH value of the medium changed to 6.94 ± 0.07 after release of SUL-MEG hydrogel. Near a neutral environment, the carboxyl group of SUL underwent complete proton dissociation, transforming from the -COOH to the negatively charged carboxylate group (-COO^−^). In addition, the secondary amino group of MEG underwent complete protonation, losing one H^+^ from the neutral -NH- group and transforming into a positively charged protonated secondary amino group (-NH_2_^+^). Thus, the essence of such complexation in deionized water (a neutral environment) might be the electrostatic interaction (i.e., ionic interaction) between -COO^−^ of SUL and the positively charged group of MEG [35,36].

## 3. Conclusions

Recent studies on pharmaceutical hydrogels have mainly focused on developing macromolecule-loaded gels by incorporating polymeric materials (e.g., starches, celluloses, and synthetic polymers), along with explorations into their formation mechanisms and multi-domain applications. In contrast, research dedicated to small-molecule drug-based hydrogels remains comparatively limited. In the present study, a novel SUL-MEG hydrogel was successfully constructed via straightforward mixing of SUL and MEG in a small volume of deionized water. Its formation mechanism was ascribed to the excellent miscibility of both components and self-assembly facilitated by intermolecular interactions. The resulting SUL-MEG hydrogel not only significantly improved the apparent solubility of SUL, but also achieved accelerated release kinetics while sustaining a prolonged supersaturated state. Furthermore, the solubilization mechanism of this hydrogel system was comprehensively explained by the complexation reaction identified through phase solubility analysis, coupled with the intrinsic high-energy nature of the hydrogel itself. Overall, this study elaborated on the design rationale of the SUL-MEG hydrogel and further clarified the mechanisms governing its formation and solubilization. It was confirmed that this design strategy serves as a feasible formulation approach to enhance the pharmaceutical performance of poorly soluble drugs, thereby optimizing their drug performance. In the future, such hydrogel systems might achieve customized drug release profiles (including rapid, sustained or controlled release) through the selection of hydrophilic or hydrophobic ligands in order to meet specific therapeutic requirements. However, their development is impeded by critical challenges. Moreover, the ligands should show excellent biocompatibility and safety for in vivo applications. Ligand selection is pivotal to gel formation and formulation performance, yet it remains a large trial and error due to the absence of systematic guiding principles. Additionally, unlike the long chains of polymeric materials, the short molecular chains of small-molecule hydrogels are susceptible to packing and rearrangement during processing or storage, which may induce recrystallization and degradation of the gel structure.

## 4. Materials and Methods

### 4.1. Materials

**Figure 5:**
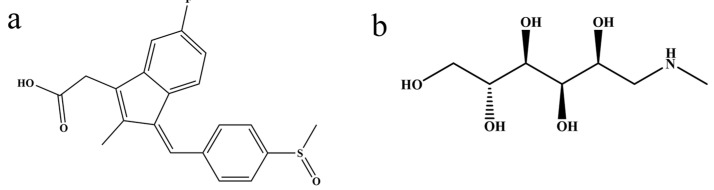
Molecular structural formulas of (**a**) SUL and (**b**) MEG.

Sulindac (SUL) and meglumine (MEG) (Figure 5) were purchased from Aladdin Biochemical Technology Co., Ltd. (Shanghai, China), with a purity of over 99%. Acetonitrile and methanol were sourced from TCI Chemicals Co., Ltd. (Shanghai, China). Additionally, phosphoric acid was procured from Sigma-Aldrich Trading Co., Ltd. (Shanghai, China). Deionized water was purified using a Milli-Q purification system (Millipore Corporation, Burlington, MA, USA).

### 4.2. Miscibility Analysis Between SUL and MEG

The miscibility of SUL and MEG was evaluated via the Hansen solubility parameter (δ) using Molecular Modeling Pro software (Version 6.3.3, Norgwyn Montgomery Software Inc., North Wales, PA, USA). The difference in solubility parameters (Δδ) between the two components serves as a critical indicator for predicting miscibility [20,21]. δ values were determined through the “group contribution method”, which breaks down δ into three constituent parts: the hydrogen bonding solubility parameter (δ_h_), polar solubility parameter (δ_p_), and dispersion solubility parameter (δ_d_) (Equation (7)). Specifically, these calculations are based on Equation (8), where F_di_, F_pi_, and E_hi_ represent the group contribution values related to dispersion forces, polarity, and hydrogen bonding, respectively, while V denotes the group contribution value for molar volume.

(7) $$\delta^{2}=\delta_{d}^{2}+\delta_{p}^{2}+\delta_{h}^{2}$$

(8) $$\delta_{d}=\frac{\sum F_{di}}{V},\delta_{p}=\frac{\sqrt{\sum F_{pi}^{2}}}{V},\delta_{h}=\sqrt{\frac{\sum E_{hi}}{V}}$$

### 4.3. Preparation of SUL-MEG Hydrogel

In the present work, a facile blending strategy was adopted to construct such a binary small-molecule hydrogel system composed of SUL and MEG in deionized water. The formation procedure initiated with the accurate weighing of SUL and MEG at an equimolar ratio, with the total mass of the two components fixed at 2000 mg. Thereafter, the weighed powder blend was transferred into a mixing vessel and subjected to constant-speed oscillation for 20 min, so as to achieve thorough and homogeneous mixing and yield the physical mixture (denoted as SUL-MEG PM). Subsequently, 1000 mg portions of the as-prepared SUL-MEG PM were separately weighed into 10 mL transparent glass vials. After that, 200 μL of deionized water was pipetted into each vial, followed by 15 min of shaking treatment. Through the above-described operation, the SUL-MEG composite was likely to undergo self-assembly and form a homogeneous hydrogel system.

### 4.4. Characterization of SUL-MEG Hydrogel

#### 4.4.1. SEM

The microscopic morphology of SUL-MEG hydrogel was investigated via scanning electron microscopy (SEM, Model TM4000, Hitachi Ltd., Tokyo, Japan). Before testing, the hydrogel sample was subjected to fixation to maintain its structural integrity. Subsequently, a thin conductive gold layer was deposited on the sample surface by gold sputtering. For the SEM measurement, the instrumental parameters were optimized to the following settings: a probe current of 20 μA and an acceleration voltage of 15 kV.

#### 4.4.2. Rheological Measurement

The viscoelastic property of SUL-MEG hydrogel was assessed using a rotational rheometer (Model MCR 702e, Anton Paar Ltd., Graz, Austria). A parallel plate geometry with a 20 mm diameter was adopted for the experimental setup. The gap between the two parallel plates was fixed at 1.0 mm, and all measurements were implemented under a temperature-controlled condition of 25 °C. Two specific testing protocols were performed: a strain sweep test at a constant frequency of 1 Hz, with the strain amplitude ranging from 0.1% to 100%; and a frequency sweep test at a fixed strain of 3%, covering a frequency interval of 0.1~100 rad/s. These assays were designed to determine two core viscoelastic parameters of the hydrogel, namely the storage modulus (G′) and loss modulus (G″). Specifically, G′ reflects the elastic behavior of the hydrogel, while G″ is indicative of its viscous response.

#### 4.4.3. Storage Stability

The prepared SUL-MEG hydrogel was sealed in an airtight manner within 10 mL glass vials, so as to avoid water evaporation and external contamination, followed by storage at ambient temperature (25 °C). For the assessment of storage stability of the SUL hydrogel, rheological measurements were performed on different days to analyze the alterations in its viscosity.

#### 4.4.4. DSC

The thermal behavior of SUL-MEG xerogel was evaluated by differential scanning calorimetry (DSC 5+, Mettler Toledo Ltd., Zurich, Switzerland). Specifically, the SUL-MEG xerogel was obtained through freeze-drying of the corresponding SUL-MEG hydrogel. For the DSC assay, about 5 mg of the SUL-MEG xerogel sample was accurately weighed out and loaded into airtight standard DSC sample crucibles. Subsequently, the sample was subjected to a linear heating program from 25 °C to 240 °C at a constant ramp rate of 10 °C·min^−1^, with the whole test procedure carried out under a nitrogen atmosphere to ensure inert conditions.

#### 4.4.5. PXRD

The PXRD pattern of SUL-MEG xerogel was acquired using an Aeris X-ray diffractometer manufactured by Malvern Panalytical Ltd. (Worcestershire, UK). For the assay, the SUL xerogel sample was placed onto the sample stage of the instrument, followed by scanning within a 2θ range of 5~40° at a step increment of 0.02°. To ensure stable X-ray emission and credible detection results, the experimental parameters were kept constant throughout the entire test: the voltage was fixed at 40 kV, and the current was maintained at 40 mA.

#### 4.4.6. FTIR

The FTIR spectrum of SUL-MEG xerogel was obtained using a Bruker INVENIO spectrometer (Bruker Ltd., Ettlingen, Germany). In the measurement process, the SUL xerogel sample was placed in the spectrometer’s sample chamber, followed by spectral scanning across the wavenumber range of 4000~500 cm^−1^. The assay was performed at a spectral resolution of 4 cm^−1^, with 32 scans accumulated to ensure the acquisition of high-quality spectral data.

### 4.5. SUL-MEG Hydrogel from the Molecular Dynamics Perspective

Small-molecule hydrogels are formed through the spontaneous self-assembly of molecules, a process primarily fueled by intermolecular interactions. Under defined environmental conditions, these weak attractive forces guide the ordered aggregation of molecules into well-organized supramolecular architectures, endowing the resulting hydrogels with unique physicochemical properties. Binding energy (E_bind_) refers to the energy associated with intermolecular interactions between different components of the system. It acts as a quantitative metric for evaluating the interaction intensity among constituent molecules: a higher absolute value signifies more robust intermolecular binding and superior thermodynamic stability of the assembly system [20,21,27,41]. A comprehensive protocol for the computation of E_bind_ based on molecular dynamics simulations was elaborated in Section S3 of the Supplementary Material.

### 4.6. Solubility of SUL-MEG Hydrogel

To assess the solubilizing capacity of SUL-MEG hydrogel, an apparent solubility test was conducted in accordance with standardized experimental protocols. An excess amount of each test sample (crystalline SUL, SUL-MEG PM, and SUL-MEG hydrogel) was separately placed into 10 mL centrifuge tubes, followed by the addition of 5 mL deionized water to trigger the dissolution process. Namely, an excess amount of the sample was added to the deionized water to ensure that undissolved samples remained at the final equilibrium. The prepared samples were incubated in a thermostatic shaker at a temperature of 37 °C and a shaking speed of 200 rpm for 24 h, which was sufficient to achieve solubility equilibrium (*n* = 3). After the incubation period, the supernatants were collected and filtered through a 0.22 μm polyethersulfone membrane for the removal of undissolved particulates. Subsequently, a 2 mL aliquot of the clarified filtrate was mixed with an equal volume of methanol to obtain the analytical solution, and the subsequent detection was carried out via high-performance liquid chromatography (HPLC), as detailed in Section S4 of the Supplementary Material.

### 4.7. In Vitro Release of SUL-MEG Hydrogel

#### 4.7.1. In Vitro Cumulative Release

The cumulative release behaviors of SUL from crystalline SUL, SUL-MEG PM, and SUL-MEG hydrogel were assessed by means of a dissolution tester (Model RCZ-12A, supplied by Huanghai Pharmaceutical Inspection Instrument Co., Ltd., Shanghai, China). All test samples were dosed at an amount equivalent to 2.1 mg of SUL. The in vitro release assays were performed under the following experimental conditions: a paddle rotation speed of 100 rpm, 900 mL of deionized water as the dissolution medium, and a thermostatically controlled environment at 37 °C; each group of experiments was replicated three times to ensure data reliability. At preset time points, 2 mL aliquots of the release medium were withdrawn and immediately replenished with an equal volume of pre-heated fresh medium, so as to maintain a constant system volume and temperature throughout the experiment. The harvested medium samples were first filtered through a 0.22 μm polyethersulfone membrane and then subjected to a 1:1 dilution with methanol as the diluent. Subsequently, the SUL concentration in the processed samples was quantified utilizing the HPLC system.

#### 4.7.2. In Vitro Supersaturation Release

The supersaturation release behaviors of crystalline SUL, SUL-MEG PM, and SUL-MEG hydrogel (dosed to an equivalent of 80 mg SUL) were assessed under the non-sink condition. Release experiments were carried out in 200 mL deionized water maintained at 37 °C, with constant stirring at 100 rpm (*n* = 3). The hydrogel sample was encapsulated in dialysis bags with a molecular weight cut-off of 14,000 Da. At preset time points, 2 mL portions of the release medium were collected, and an equal volume of fresh and pre-warmed medium was promptly added to maintain a consistent total volume. Each collected sample was first filtered via a 0.22 μm polyethersulfone membrane, followed by mixing with an equal volume of methanol. Quantification of SUL concentration was conducted via the HPLC system.

### 4.8. Complexation Between SUL and MEG by Phase Solubility Study

The phase solubility method was employed to investigate whether complexation occurred between SUL and MEG in aqueous solutions. Excess crystalline SUL was individually added to MEG aqueous solutions with gradient concentrations (*n* = 3). Subsequently, these samples were placed in a thermostatic shaker, where they were oscillated at 37 °C and 200 rpm for 24 h. After this oscillation treatment, 2 mL of the supernatant was carefully pipetted and filtered through a 0.22 μm polyethersulfone membrane. The concentration of SUL in the resulting filtrate was determined via the HPLC method.

## Data Availability

Data will be made available on request.

## Supplementary Materials

The following supporting information can be downloaded at https://www.mdpi.com/article/10.3390/gels12030198/s1. Figure S1: Microscopic structure of SUL-MEG hydrogel observed under the cryogenic state; Figure S2: Variation curves of dynamic property for SUL-MEG-H_2_O system during MD simulation under NPT ensemble: (a) Temperature, (b) Density and (c) Energy; Figure S3: Concentration-time curves of crystalline SUL and SUL-MEG hydrogel in pH 1.2 HCl buffer; Table S1: Theoretical δ calculation of SUL and MEG; Table S2: The viscosity values of SUL-MEG hydrogel at 25 °C (Pa·s) (cP ± SD, *n* = 3); Table S3: Binding energy (E_bind_) of binary and ternary systems of SUL, MEG and H_2_O at 25 °C; Table S4: Comparison of dissociation properties for SUL and MEG; Table S5: Change in pH values of deionized water before and after the supersaturated release of crystalline SUL and SUL-MEG hydrogel (*n* = 3).
