# Supplementary material for: Formation and Release Enhancement of a Novel Small-Molecule Hydrogel Containing Sulindac and Meglumine
Source: Gels. 2026 Feb 27;12(3):198. doi: 10.3390/gels12030198 (PMC13024745; doi:10.3390/gels12030198)
Supplement: Supplementary file 1 [file gels-12-00198-s001.zip › gels-4145996-supplementary.pdf]

Formation and release enhancement of a novel small-molecule hydrogel containing sulindac and meglumine

Jiaxin Chen†, Baimin Niu†, Huizhen Sun, Weitao Fang, Mingjun Li, Xinru Lu, Jue Wang, Jiawei Han*, Xiaoqian Liu*

School of Pharmacy & School of Biological and Food Engineering, Changzhou University, Changzhou, 213164, P.R., China

† These authors contributed equally to this work.

Corresponding authors and address for reprint:

*Corresponding authors

Prof. Jiawei Han

School of Pharmacy & School of Biological and Food Engineering, Changzhou University, Changzhou;

Tel.: +86 159 5197 9228;

E-mail: hanjiawei329@cczu.edu.cn

Prof. Xiaoqian Liu

School of Pharmacy & School of Biological and Food Engineering, Changzhou University, Changzhou;

Tel.: +86 151 8978 8736;

E-mail: chmliux@cczu.edu.cn

S1. Theoretical miscibility of SUL and MEG

Table S1. Theoretical δ calculation of SUL and MEG.

**Component**	**δ (MPa $^{1/2}$)**	**Δδ (MPa $^{1/2}$)**
SUL	23.74	2.15
MEG	25.89	

S2. Microstructure and storage stability of SUL-MEG hydrogel

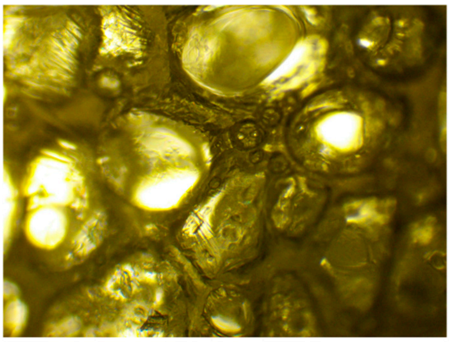

Figure S1. Microscopic structure of SUL-MEG hydrogel observed under the cryogenic state.

Table S2. The viscosity values of SUL-MEG hydrogel at 25 °C (Pa·s) (cP ± SD, *n* = 3)

Hydrogels	0 day	15 days	30 days
SUL-MEG hydrogel	5733.59 ± 42.18	5705.80 ± 13.75	5721.18 ± 33.10

S3. Formation of SUL-MEG hydrogel by molecular dynamics simulation

The small-molecule hydrogel was formed by self-assembly of molecules due to non-covalent interactions or dynamic covalent bonds [22]. Molecular dynamics simulation was used to calculate the binding energy (E_{bind}) between components and further explore such formation mechanism of SUL-MEG hydrogel. The E_{bind} was defined as the intermolecular interaction energy between different components (Equation S1). E_{bind} obtained from molecular dynamics simulation at the molecular level, is frequently used to assess the interactions of two or multiple components, such as in the fields of co-crystal, co-amorphous and amorphous solid dispersion

formulations [20, 21]. The Forcite module of Materials Studio software was used to carry out molecular dynamics simulation at 298 K (i.e., the preparation temperature of 25 °C).

$$E_{bind} = -[E_{total} - (E_{layer(1)} + E_{layer(2)} + \dots + E_{layer(n)})] \qquad (S1)$$

E_{bind}: the binding energy of the hydrogel system

E_{total}: the total energy of the entire equilibrium structure

$E_{layer(1)}$, $E_{layer(2)}$ and $E_{layer(n)}$: the total energy of the first, second and n layers

(1) Model construction of SUL-MEG-H_2O cell

Step 1: The Forcite module of Materials Studio software was used for the geometric optimization of SUL, MEG and H_2O molecules to minimize their energy. The relevant parameters of the molecular dynamics simulation included Task (Geometry Optimization), Quality (Fine), Forcefield (COMPASS II) and Charges (Forcefield assigned). Besides, Electrostatic and van der Waals were set to Atom based.

Step 2: The optimized molecules of SUL, MEG and H_2O were selected to build the SUL-MEG-H_2O cell, SUL-H_2O cell and MEG-H_2O cell by the Amorphous Cell module of Materials Studio software. The molecular ratios of SUL, MEG and H_2O were based on the molar mass of actual preparation. The relevant parameters of molecular dynamics simulation included Task (Construction), Quality (Fine), Forcefield (COMPASS II), Charges (Forcefield assigned), Electrostatic force (Ewald) and van der Waals (Atom based).

(2) Molecular dynamics simulation details

The Forcite module of Materials Studio software was used to simulate the preparation process of the constructed SUL-MEG-H_2O cell. The relevant parameters of molecular dynamics simulation included Task (Dynamics), Quality (Fine), Ensemble (NPT), Temperature (298 K, i.e., preparation temperature of 25 °C), Pressure (0.0001 GPa), Total simulation time (200 ps), Time step (1 fs), Number of steps (2×10^5). Other

parameters included Thermostat (Andersen), Barostat (Berendsen), Forcefield (COMPASS II), Charges (Forcefield assigned), Electrostatic force (Ewald) and van der Waals (Atom based) [26, 27]. The equilibrium determination can be based on the changes of the system's temperature, density, and potential energy over time. Generally, when the fluctuation range of the data is within 5% ~ 10%, it can be considered that the thermodynamic equilibrium state has been reached. As shown in Figure S2, SUL-MEG-H_2O system reached a balanced state at approximately 40 ps in the simulation time. Finally, the equilibrium layered structures of SUL-MEG-H_2O cell, SUL-H_2O cell and MEG-H_2O cell could be obtained to calculate their binding energy (E_{bind}) (Table S3).

Table S3. Binding energy (E_{bind}) of binary and ternary systems of SUL, MEG and H_2O at 25 °C.

Sample	Total energy	Layer (1)	Layer (2)	Layer (3)	E_{bind}
**SUL/MEG/H_2O**					
SUL/MEG/H_2O	1498.748	536.604	2113.916	-962.254	189.518
**SUL/H_2O**					
SUL/H_2O	-554.876	528.807	-975.693		107.99
**MEG/H_2O**					
MEG/H_2O	997.497	2105.76	-985.16		123.103

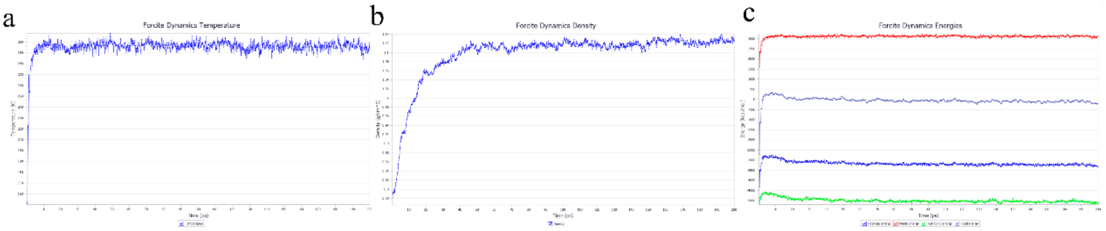

Figure S2. Variation curves of dynamic property for SUL-MEG-H_2O system during MD simulation under NPT ensemble: (a) Temperature, (b) Density and (c) Energy.

S4. Content determination of SUL

Quantitative analysis of SUL concentration was carried out *via* a high-performance liquid chromatography (HPLC) system (Nexera LC-40, Shimadzu Co. Ltd., Japan) fitted with a Sharpsil-U C18 chromatographic column (4.6 mm × 250 mm, 5 μm particle diameter). Chromatographic separation was accomplished with a mobile phase consisting of acetonitrile and 0.3% aqueous phosphoric acid solution (v/v, 50:50) at a

steady flow rate of 1 mL/min. Meanwhile, the column temperature was kept at 35 °C, and detection was conducted at a wavelength of 320 nm to ensure optimal sensitivity for SUL quantification.

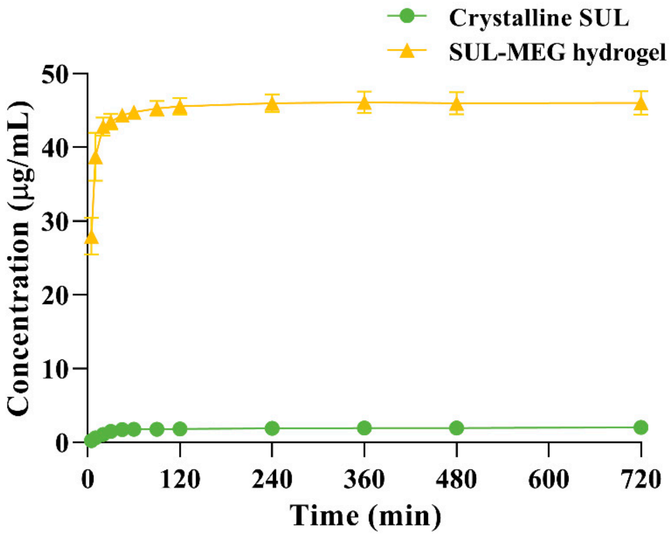

Figure S3. Concentration-time curves of crystalline SUL and SUL-MEG hydrogel in pH 1.2 HCl buffer.

S5. Analysis of complexation essence

Table S4. Comparison of dissociation properties for SUL and MEG.

**Substance name**	**Dissociable functional group(s)**	**pKa value (25°C)**	**Charge state at different pH values**
SUL	Carboxyl group (-COOH)	4.7	pH < 4.7: Neutral (exists as -COOH)<br>pH ≈ 4.7: Coexistence of -COOH/-COO⁻<br>pH > 4.7: Negatively charged (exists as -COO⁻)
MEG	Secondary amino group (-NH-)	9.39~9.6	pH < 9.39: Positively charged (exists as $-NH_2^+$)<br>pH ≈ 9.39~9.6: Coexistence of -NH/$-NH_2^+$<br>pH > 9.6: Neutral (exists as -NH-)

Table S5. Change in pH values of deionized water before and after the supersaturated release of crystalline SUL and SUL-MEG hydrogel (*n* = 3).

Group	Initial pH value	pH value after supersaturated release
Deionized water	7.08 ± 0.01	/
Crystalline SUL	/	6.56 ± 0.21
SUL-MEG hydrogel	/	6.94 ± 0.07
